# Customer churn modeling in telecommunication using a novel multi-objective evolutionary clustering-based ensemble learning

**DOI:** 10.1371/journal.pone.0303881

**Published:** 2024-06-06

**Authors:** Kaveh Faraji Googerdchi, Shahrokh Asadi, Seyed Mohammadbagher Jafari

**Affiliations:** 1 Faculty of Management and Accounting, College of Farabi, University of Tehran, Tehran, Iran; 2 Faculty of Engineering, College of Farabi, University of Tehran, Tehran, Iran; Institute of Management Sciences Peshawar, PAKISTAN

## Abstract

Customer churn prediction is vital for organizations to mitigate costs and foster growth. Ensemble learning models are commonly used for churn prediction. Diversity and prediction performance are two essential principles for constructing ensemble classifiers. Therefore, developing accurate ensemble learning models consisting of diverse base classifiers is a considerable challenge in this area. In this study, we propose two multi-objective evolutionary ensemble learning models based on clustering (MOEECs), which are include a novel diversity measure. Also, to overcome the data imbalance problem, another objective function is presented in the second model to evaluate ensemble performance. The proposed models in this paper are evaluated with a dataset collected from a mobile operator database. Our first model, MOEEC-1, achieves an accuracy of 97.30% and an AUC of 93.76%, outperforming classical classifiers and other ensemble models. Similarly, MOEEC-2 attains an accuracy of 96.35% and an AUC of 94.89%, showcasing its effectiveness in churn prediction. Furthermore, comparison with previous churn models reveals that MOEEC-1 and MOEEC-2 exhibit superior performance in accuracy, precision, and F-score. Overall, our proposed MOEECs demonstrate significant advancements in churn prediction accuracy and outperform existing models in terms of key performance metrics. These findings underscore the efficacy of our approach in addressing the challenges of customer churn prediction and its potential for practical application in organizational decision-making.

## Section 1: Introduction

The world’s population in 2020 was 7.8 billion [1], of which 67% were subscribers of mobile services by the end of 2019. On top of this, 600 million more subscribers are expected by 2025 [2]. Additionally, the mobile phone penetration rate in big cities of some countries was above 100%, which means that the number of subscribers was more than the residents of those cities [3]. Accordingly, the telecommunications market, especially in large cities, is competitive and saturated. In a market like this, organizations’ competitiveness depends on the number of subscribers, so suppliers compete for more customers [4].

The presence of multiple suppliers in the mobile phone market has given customers the option to choose between different providers if they are not satisfied with a given provider’s services [5]. This phenomenon, known as customer churn, is one of the primary reasons for telecom companies’ failure. Many organizations lose 25 percent of their subscribers annually, costing them between $ 2 billion and $ 4 billion [6]. A successful company such as Verizon lost 1.22% of its customers in 2018 [7]. Therefore, the lower the customer churn, the more profitable the organization is. As a result, companies try to use accurate tools to predict customer churn and prevent it by adopting appropriate strategies [8].

The concept of churn is studied in many industries. Some of these studies identify factors that influence churn, but most research on churn focuses on improving churn prediction. In these researches, different tools are used for churn prediction. Data mining and machine learning are some of these tools. Data mining has become one of the most effective methods for predicting customer attitude [9]. In previous studies, various supervised learning techniques such as decision tree [10], neural networks [11] and support vector machine [12] were employed to predict churn.

Ensemble learning is a popular approach in machine learning, in which several base classifiers will train on a training data of a particular problem, and they are then combined to create a model that its’ classification error will be less than each base classifier [13]. Diversity and classification performance are two main principles in building ensemble classifiers [14]. The diversity between base classifiers is essential in creating ensemble learning models because, without diversity, the ensemble classifier will not differ from the individual classifier [13].

There are various methods for generating ensemble models, each of which somehow ensures diversity between base classifiers. Using clustering; to creating diverse subsets of data and train classifiers with different structures by each cluster, is an efficient approach for ensemble classifier creation. [15, 16]. It is critical in this method to select a set of diverse classifiers that together will form an accurate ensemble model. The diversity and performance of ensemble classifiers are two main factors in this regard. Different diversity measures are introduced in [17]. These measures evaluate the diversity of two classifiers based on their classification error differences. In a sense that if two classifiers’ mis predict the same sample but have different classifications about it, then they are considered to be the same. Moreover, performance evaluation metrics are presented in [18]. A vital issue in classification performance evaluation is the class imbalance problem [19]. In the class imbalance problem, identifying minority class samples are more challenging than majority ones [20]. In ensemble models, there is an inverse relationship between diversity and accuracy, and the right balance between them produces an ensemble with optimal performance [17].

The main contribution of this paper is to present an efficient model for predicting customer churn, which uses a multi-objective optimization method to select a set of base classifiers whose combined model not only has a maximum diversity but also can accurately classify the samples. Additionally, a novel diversity measure is proposed that evaluates the diversity of two classifiers based on the differences between their predictions. Hence, if two classifiers predict different classes about a sample, the proposed measure will evaluate that difference. An additional metric is also proposed to solve the data imbalance problem in the proposed model.

A layered base clustering method is used to create diversity in proposed model and divide the training data into subspaces. This method divides the training set into heterogeneous and diverse clusters. Then, five classifiers with different structures will train on each cluster. Finally, using the non-dominated sorting genetic algorithm II (NSGA-II), considering the two goals of diversity and accuracy, a set of classifiers will be selected among the trained classifiers. Although the ensemble model created from the combination of these classifiers has a high degree of diversity, it can also classify data accurately.

The rest of this paper is organized as follows. Section 2 reviews previous churn studies and state-of-the-art approaches for ensemble classifier generation, Section 3 discusses the proposed method and proposed metrics. Section 4 states the experimental framework, results, and conducted comparisons, and finally, Section 5 presents conclusions.

## Section 2: Literature review

Customer churn is one of the most important issues companies face because it directly impacts their growth and profitability. Therefore, companies have carried out various activities to identify and predict customer churn. This section reviews the related literature to customer churn and ensemble learning.

### Customer churn

The conducted studies examine the churn concept from two perspectives. The first perspective tries to identify customer churn reasons, which can be divided into three categories. The first category of these studies examines organizational factors such as organizational strategies [21], product characteristics [22] or organizational factors that cause customer dissatisfaction [23]. The second category investigates behavioral factors such as pre-churning behaviors that can be identified based on the change of customers’ position in their social networks [24] or customers’ daily behaviors that can be used to identify churning customers [4] quickly. The third category is those studies that seek social factors that affect the churning and identification of vital nodes in social networks [25].

The second perspective of churn studies tries to help organizations retain their customers by providing accurate and efficient methods to predict churn. Numerous studies have been presented with the aim of providing an effective and accurate method for predicting customer churn in the telecommunications industry [5, 26]. Data mining techniques used in churn prediction include decision tree [27], logistic regression [28], support vector machine [29], artificial neural networks [30], k nearest neighbor [31], and hybrid techniques [32].

### Ensemble learning

Ensemble learning is one of the latest approaches in data mining and machine learning. Ensemble learning is a machine learning paradigm in which several base classifiers are trained by the training data of a particular problem, and then combined to produce a model where the classification error expect to be lower than that of the individual base classifiers [33]. Among the most popular ensemble models are Bagging [34], Boosting [35] and Random Forest [36].

Various studies on churn prediction have been done, which indicate that ensemble learning models are superior to classical models [37]. In churn studies, ensemble learning models such as Rotation Forest, RotBoost [38, 39] and hybrid models [3] have been used to predict customer churn. A critical point in ensemble learning is to achieve more accurate prediction by combining a set of diverse classifiers [40]. In this regard, ensemble learning methods can be examined from two perspectives. The first perspective examines the functionality of ensemble learning models, and the second perspective investigates the creating approaches of ensemble learning models [14].

From the first perspective, ensemble learning models can be classified into parallel and sequential categories. In parallel ensemble learning models, each base classifier of the ensemble learning model will train independently, while in the sequential ensemble learning models, each base classifier’s output affects the training and performance of the next classifier. Among the most popular parallel ensemble learning models, one can mention bagging [34] and random forest [36]. Sequential ensemble learning models are boosting-base models, in which when training a new base classifier, the focus is on samples that were not classified correctly in the previous steps. The weight of each sample will determine how much base classifiers will focus on it. In the first stage, all samples’ have the same weight, and with each iteration, the weight of incorrectly classified samples increases. Some Popular sequential ensemble learning algorithms include AdaBoost [41] and Gradient Boosting [42].

According to the second perspective, ensemble learning models can be classified into four categories based on their construction process. The first category of this perspective; includes methods that create diversity among base classifiers by applying changes to the base classifiers algorithms when creating an ensemble classifier. One way to do this is to change the base classifiers’ parameters [43], and another method is to use heterogeneous classifiers [16] as base classifiers in the ensemble learning model.

The second category is those ensemble classifiers that create diversity among base classifiers by making changes to the input data. These changes will accomplish in different ways. One way is input data segmentation, which will perform by some techniques such as clustering as in [44], random sampling as in bagging-based algorithms [45], or partitioning training samples based on more informative tuples as in AdaBoost-based algorithms [46]. Another way is to divide properties of the dataset into separate subsections like what happens in the Random Subspace Ensemble [47]. In [48], a new ensemble learning approach is introduced in which feature space is divided into mutually distinct regions. [49] also introduces the Attribute bagging method in which an attempt is made to improve the accuracy of ensemble classifier by random sampling of features.

The third category is hybrid ensemble classifiers that use at least two different strategies to produce an ensemble learning model. One of the most famous hybrid ensemble classifiers is the random forest [36]. RotBoost [50] is also an example of hybrid models, which is a combination of Rotation Forest and AdaBoost. In [51], the authors proposed the nonlinear boosting projections method to produce an ensemble learning model that combines two methods of boosting and random subspace.

The fourth category is the methods that form an ensemble learning model by optimization concepts. For example, in [52], the authors used genetic algorithms (GAs) to create ensemble classifications. In [53], the authors also introduced a mechanism for learning the optimal classification for an ensemble system by considering three objectives; the number of correctly classified samples, the number of selected features and the number of selected classifiers. In [54], researchers used particle swarm optimization (PSO) as a model selection tool to select the best set of base classifiers to produce an ensemble model. [15] also presented a hierarchical optimization framework based on divide-and-conquer for ensemble classification learning. In this research, the accuracy of classification in each class is calculated as a separate objective function.

As mentioned, one way to create diversity in ensemble learning models is to divide the input data by clustering. In [44], researchers propose a new approach to producing and training ensemble learning models. This approach is based on the production of atomic and non-atomic clusters at different levels. In [55], researchers also introduce the Non-Uniform Layered Cluster Oriented Ensemble Classifier, in which the dataset will divide into a several clusters at each level, and a group of classifiers will train on each cluster. In this paper, the authors use GA to find the optimal number of layers and clusters. In [56], the researchers propose a cluster-based ensemble classification generation method and a genetic algorithm-based approach to parameter optimization. [57] also presented a hierarchical ensemble classification algorithm based on clustering confidence vectors. In [58], researchers used a multi-objective evolutionary algorithm to find the optimal combination of layers and the number of clusters in the Non-Uniform Layered Cluster Oriented Ensemble Classifier method. In [16], in addition to introducing a new diversity measure, the authors introduced an Incremental Layered Classifier Selection approach that incrementally selects the base classifiers from the base classifier pool.

The literature review highlights several significant challenges in the fields of customer churn prediction and ensemble learning. From the perspective of customer churn, studies have focused on identifying churn reasons and developing effective prediction methods. However, challenges remain in accurately predicting churn due to complex factors such as organizational, behavioral, and social influences. In the realm of ensemble learning, the main challenge lies in achieving diversity among base classifiers while maintaining high prediction accuracy. Current methodologies employ various strategies, including parallel and sequential ensemble models, hybrid approaches, and optimization-based methods. Nonetheless, selecting the appropriate ensemble, creating diversity, and ensuring desired performance remain critical challenges. Addressing these challenges is vital for developing robust ensemble learning models capable of accurately predicting customer churn in dynamic and competitive markets.

According to the literature mentioned above, there are three main issues in creating ensemble classifiers; selecting the appropriate ensemble, creating diversity in ensemble classifier and the desired performance of ensemble classifier. In this study, a multi-objective ensemble learning model based on clustering is presented to address these issues. The contributions of the proposed method are as follows:

First, using layered clustering, the training dataset is divided into different clusters. In each layer, the training set is divided into several clusters by the K-means clustering algorithm, in which the number of clusters in each layer must increase to one more. The clustering process continues as long as the upper bound of clustering allows the algorithm. Upon completion of the clustering process, repetitive clusters will remove, to increase the diversity among clusters. Additionally, those clusters that contain samples of just one class will be eliminated from the set of clusters. Then, a set of ANN, KNN, DT, BN and SVM classifiers are trained by each cluster, and the trained classifiers are stored in a given space.

Second, the trade-off optimization between accuracy and diversity (for Pareto-front identification) has been proposed. In this step, a novel measure is presented to calculate the diversity between base classifiers using their predictions, not their classification error. The advantage of using this measure is to find completely different classifiers among the set of base classifiers.

Third, in order to overcome the imbalance problem, a new objective function is introduced, which can be used to evaluate the performance of a classifier in all classes simultaneously. This objective function helps the proposed method select a set of base classifiers, whose ensemble classifier resulting from their combination by majority voting, has the optimal performance in classifying samples of all classes. In the next section, the proposed method is discussed. In Table 1, we provide a comprehensive summary of state-of-the-art churn prediction techniques along with their performances, offering an insightful overview of the literature in this field.

**Table 1:** Literature review of papers on churn prediction in telecommunication.

Ref.	Year	Authors	Techniques	Accuracy	AUC	Recall	Specificity	Precision	F-score
[59]	2024	S. K. Wagh; A. A. Andhale; K. S. Wagh; J. R. Pansare; S. P. Ambadekar; S.H. Gawande	DT + up-sampling & Edited Nearest Neighbors	93.85	-	92.0	-	93.0	93.0
			RF + sampling & Edited Nearest Neighbors	99.09	-	100	-	98.0	99.0
[60]	2024	T. Zdziebko, P. Sulikowski, W. Sałabun, M. Przybyła-Kasperek, and I. Bąk	Mamdani model	-	-	62.9	-	80.3	70.6
[61]	2024	S. Saha; C. Saha; M. M. Haque; M. G. R. Alam; A. Talukder	SMOTEEN + ChurnNet	95.59	98.76	96.22	-	95.88	96.04
[62]	2023	I. Abdullaev; N. Prodanova; M. A. Ahmed; E. L. Lydia; B. Shrestha; G. P. Joshi; W. Cho	AIJOA-CPDE	91.41	91.41	91.41	-	97.63	94.20
[63]	2023	Y. Zhou; W. Chen; X. Sun; D. Yang	BPNN	-	-	94.0	-	94.0	93
			RF	-	-	97.0	-	97.0	97.0
			Adaboost	-	-	96.0	-	97.0	96.0
			RF-Adaboost	-	-	98.0	-	98.0	98.0
[64]	2023	L. Saha; H. K. Tripathy; T. Gaber; H. El-Gohary; El-Sayed M. El-kenawy	Extreme Randomized Tree	94.0	70.0	94.0	76.0	93.0	93.0
			XGBoost	94.0	73.0	95.0	67.0	98.0	97.00
			Gradient Boosting	94.0	74.0	96.0	70.0	98.0	97.0
			Convolutional Neural Network	99.0	99.0	99.0	99.0	99.0	99.0
[65]	2022	R Sudharsan, EN Ganesh	Swish Recurrent Neural Network	95.99	-	98.27	92.31	95.38	96.8
[66]	2022	S. F. Bilal1; A. A. Almazroi; S. Bashir; F. H. Khan; A. A. Almazroi	Clustering with Single Classifier	88.15	-	71.38	-	36.65	42.11
			Clustering with Voting Ensemble	89.58	-	88.55	-	31.49	44.18
			Clustering with Bagging	89.61	-	89.82	-	31.07	43.78
			Clustering with Stacking	89.29	-	87.66	-	30.82	40.24
			Clustering with AdaBoost	90.66	-	71.70	-	55.05	59.49
[67]	2022	S. W. Fujo; S. Subramanian; M. A. Khder	Deep-BP-ANN	86.57	86.57	94.45	-	81.59	87.55
[68]	2021	S. Wu; W.C. Yau; T.S. Ong; S.C. Chong	SMOTE + LR	74.82	84.39	78.76	-	51.74	62.43
			SMOTE + DT	76.74	82.83	72.07	-	54.97	62.26
			SMOTE + RF	76.99	83.80	73.25	-	55.14	62.86
			SMOTE + AdaBoost	77.19	84.52	73.35	-	55.44	63.11
			SMOTE + MLP	75.60	84.05	76.30	-	52.88	62.45
[69]	2021	Sebastián Maldonado, Gonzalo Domínguez, Diego Olaya, Wouter Verbeke	Logit	56.43	68.76	-	52.59	-	-
			KNN	64.07	67.68	-	65.22	-	-
			CART	57.05	64.49	-	52.92	-	-
			RF	66.24	72.33	-	66.51	-	-
			ANN	58.77	69.25	-	55.97	-	-
			SVM	64.12	70.73	-	63.88	-	-
[70]	2020	Debjyoti Das Adhikary & Deepak Gupta	AdaBoost	71.36	0.61	0.71	-	NA	NA
			Bagging	71.39	0.61	0.71	-	0.69	0.59
			Bayes Net	66.40	0.61	0.66	-	0.63	0.64
			Multilayer Perceptron	72.06	0.50	0.72	-	0.51	0.60
			Naïve Bayes	71.98	0.49	0.71	-	0.57	0.60
			Neural Network	72.15	0.50	0.72	-	0.67	0.61
			Random Forest	70.38	0.60	0.70	-	0.64	0.64
			Others …	-	-	-	-	-	-
[4]	2020	N Alboukaey, A Joukhadar, N Ghneim	CNN- Deep Learning	-	90.4	-	-	-	52.0
			LSTM-based Deep Learning	-	91.4	-	-	-	54.2
			RFM-based Random Forest	-	90.6	-	-	-	52.5
			RF-Monthly	-	85.8	-	-	-	45.6
[28]	2020	H Jain, A Khunteta, S Srivastava	Logistic Regression	85.2	71.7	85.2	-	80.6	81.0
			Logit Boost	85.2	71.8	85.2	-	80.2	80.6
[71]	2019	Mehpara Saghir; Zeenat Bibi; Saba Bashir; Farhan Hassan Khan	Deep Learning	74.25	-	69.37	-	72.28	70.80
			Neural Nets	80.54	-	75.65	-	80.03	77.78
			Auto MLP	80.58	-	76.08	-	80.02	78.00
			Bagging DL	70.76	-	67.47	-	57.84	59.60
			AdaBoost DL	73.9	-	73.74	-	70.46	72.06
			Bagging NN	79.92	-	77.3	-	81.56	79.37
			AdaBoost NN	80.02	-	77.34	-	77.49	77.41
			Bagging MLP	80.86	-	75.28	-	81.88	78.44
			AdaBoost MLP	80.08	-	75.56	-	78.03	76.78
			Majority Voting DL+NN+MLP	80.27	-	75.23	-	74.91	75.07
[26]	2019	Irfan Ullah; Basit Raza; Ahmad Kamran Malik; Muhammad Imran; Saif Ul Islam; Sung Won Kim	RF	88.8	94.7	88.8	-	89.3	88.2
			Attribute Selected Classifier	88.8	91.3	88.8	-	90.2	88.00
			Bagging +Random Tree	88.1	93.0	88.1	-	88.3	87.6
			Multilayer Perceptron	82.2	80.4	82.2	-	82.1	81.0
			Others …	-	-	-	-	-	-
[5]	2019	A. Amin, F. Al-Obeidat, B. Shah, A. Adnan, J. Loo, S. Anwar	Naïve Bayes	57	-	61.30	-	54.14	57.50
[72]	2018	J Vijaya, E Sivasankar	DT + K-means	90.32	-	95.67	07.77	-	-
			KNN + K-means	94.72	-	98.49	1.94	-	-
			SVM + K-means	93.77	-	100.0	0.00	-	-
			NB + K-means	94.14	-	99.48	0.09	-	-
			LDA + K-means	93.72	-	99.85	0.06	-	-
			DT + K-Medoids	90.43	-	95.24	09.15	-	-
			KNN + K-Medoids	94.41	-	98.99	1.99	-	-
			SVM + K-Medoids	93.32	-	100.0	0.00	-	-
			NB + K-means	93.14	-	99.48	00.14	-	-
			LDA + K-Medoids	92.21	-	99.01	00.09	-	-
[73]	2017	Ammar A.Q. Ahmed, Maheswari D.	Firefly Algorithm	86.38	-	80	-	90	93
[74]	2016	R. Yu; X. An; B. Jin; J. Shi, O.A. Move; Y. Liu	BP	63.59	-	45.76	85.43	-	-
			PSO-BP	69.64	-	51.43	87.84	-	-
			Particle Classification Optimization-based BP	73.29	-	57.07	89.52	-	-
[75]	2016	A.J. Petkovski; B.L.R. Stojkoska; K.V. Trivodaliev; S.A. Kalajdziski	Naïve Bayes	85.24	82.2	-	-	-	-
			K-Nearest Neighbors	90.59	85.7	-	-	-	-
			Logistics Regression	94.35	92.8	-	-	-	-

## Section 3: The proposed method

Numerous studies have pointed to the need for diversity in ensemble classifiers so far. There are many ways to create diversity in ensemble learning models, which the most common one is to divide the training dataset into diverse subsets and train base classifiers by each subset. One approach to creating ensemble classifiers is to cluster the dataset, train the base classifiers by each cluster, and combine the classifiers’ decisions. The selection of a set of base classifiers after clustering is an important issue in the formation of ensemble classifier by this method.

The main focus of this paper is to identify the optimal ensemble classifier through a multi-objective optimization algorithm by considering the two goals of diversity and classification accuracy. In order to accurately detect the diversity between base classifiers, a new measure is introduced that evaluates the diversity between classifiers, regardless of whether their predictions are correct or incorrect. Also, to evaluate the performance of the classifiers, a new metric is introduced that examines the classifier’s performance by considering the class imbalance problem.

**Fig 1:**
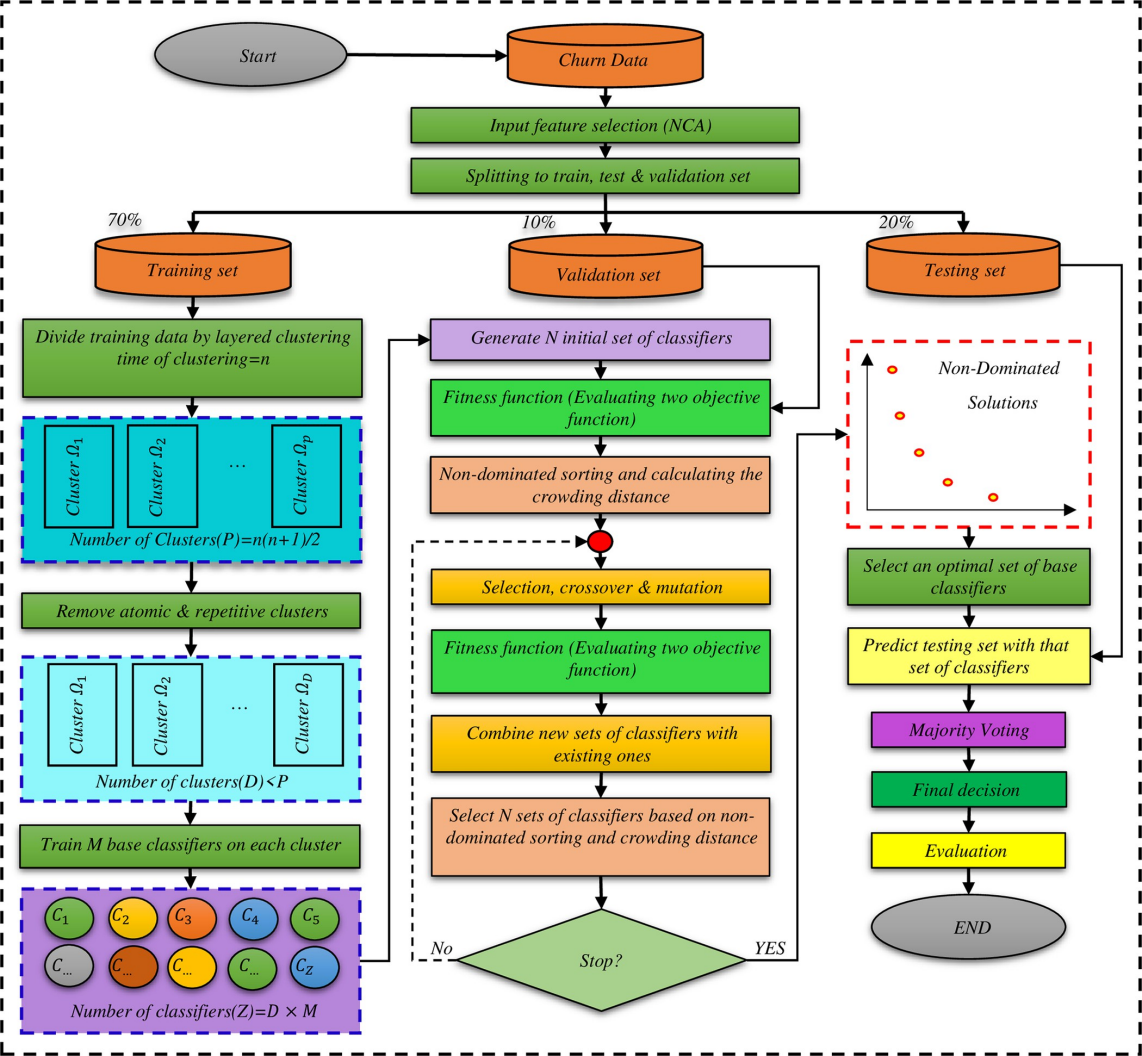
The flowchart of the proposed method.

In this paper, the objective is to simultaneously optimize the diversity and accuracy of classification to create optimal ensemble classifiers by which customer churn can be predicted with high accuracy. To do so, the NSGA-II algorithm proposed by [76], has been used. The proposed method can be implemented in two ways. In the following, first, the proposed method’s structure is described, then the coding of chromosomes, as well as two versions of the proposed method, are explained. The general flowchart of the proposed method is illustrated in Fig 1.

### Data preparation

The proposed method first reduces the input feature space by selecting a set of features that can maximize the ensemble classification’s overall accuracy. By doing so, the critical features are identified, and less important features are removed. To calculate the weight of the features, the NCA method is implemented on the dataset. According to the obtained results, some features such as ’the amount of credit charged’, ’the number of text messages sent’ and ’the type of service plan selected by the user’ are excluded from the feature set. After feature selection, to maintain the experiments in a suitable random environment, seventy percent of the dataset is randomly allocated to the training set, ten percent to the validation set, and the rest to the testing set.

### Creating search space

In the proposed method for creating the optimal ensemble classifier, the evolutionary algorithm must select the base classifiers from the set of trained classifiers whose combination by the majority vote can accurately predict the test samples. The search space of the evolutionary algorithm contains a set of such classifiers. The process of forming this space is described below.

#### Clustering

**Fig 2:**
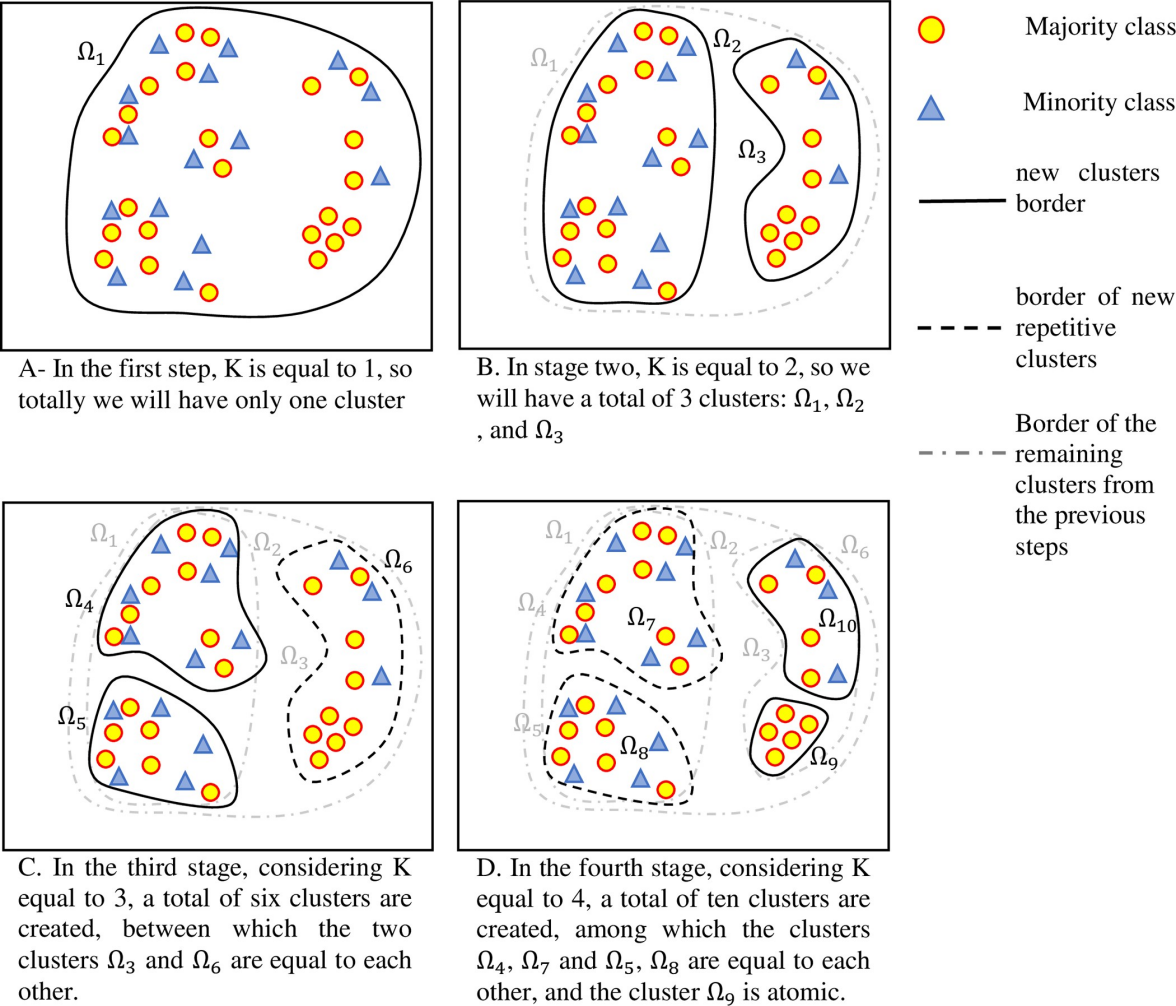
The clustering process of the training dataset.

In the proposed method, K-means is used for clustering the training dataset, and in each level, the number of clusters (K) will be one cluster more than the previous level. Finally, all the clusters created at all levels are stored in a given space for comparison and training operations. After the n^th^ level, the total number of clusters created in the storage space will be equal to n(n + 1)/2. Fig 2 shows the clustering process and the total number of clusters after four levels.

**Fig 3:**
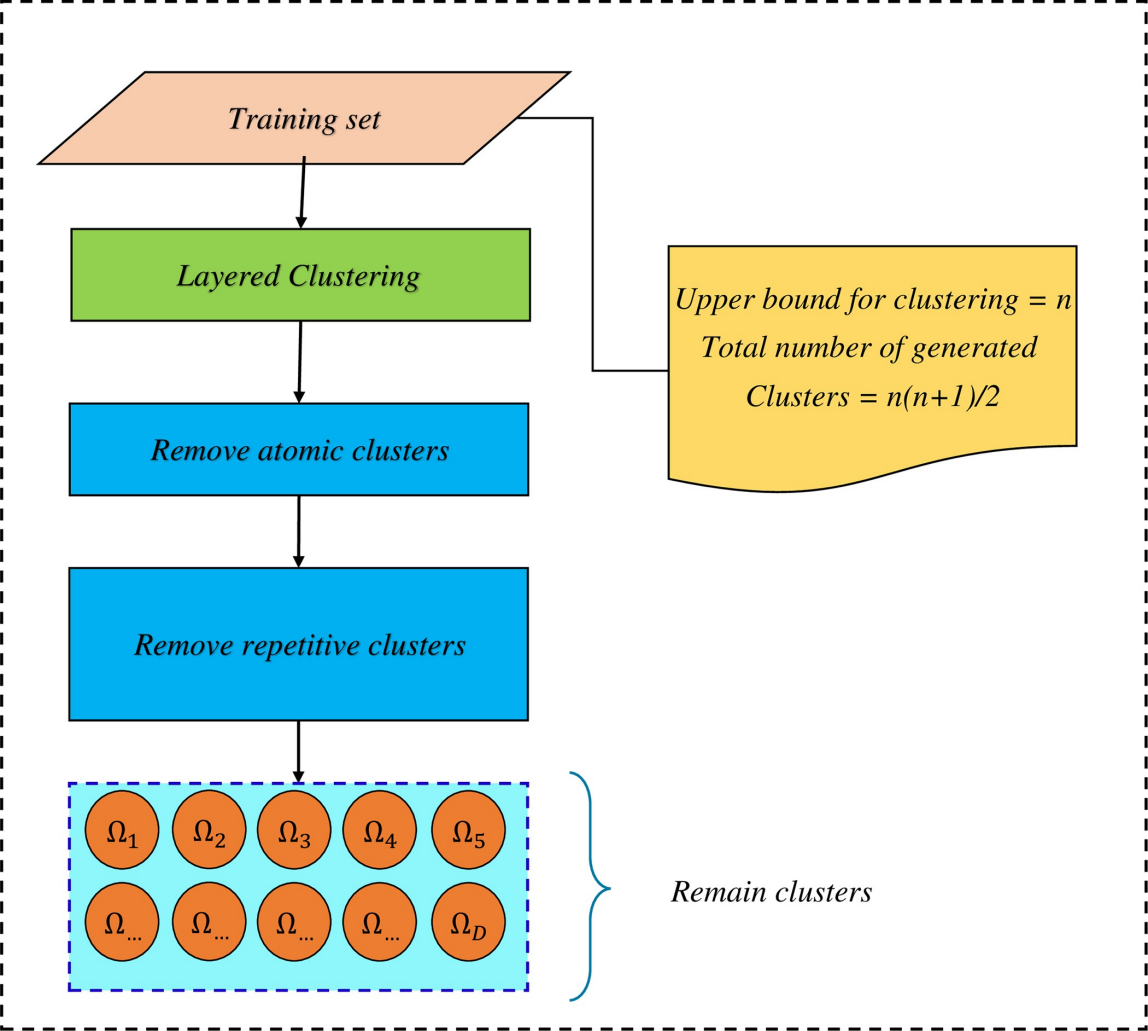
The clustering process, removing atomic clusters and removing duplicate clusters.

The problem with this clustering method is that it creates duplicate clusters at different stages. Therefore, an operator must be used to remove duplicate clusters. Also, due to the imbalance nature of the data, after clustering, some clusters will include samples that all belong to the same class. These types of clusters are known as Atomic clusters [57]. The process of training classifiers on these types of clusters is useless. Accordingly, these clusters are also removed from the cluster set. Fig 3 shows the process of clustering and removing duplicate clusters. The remaining clusters are used to train the base classifiers.

#### Training the set of base classifiers

**Fig 4:**
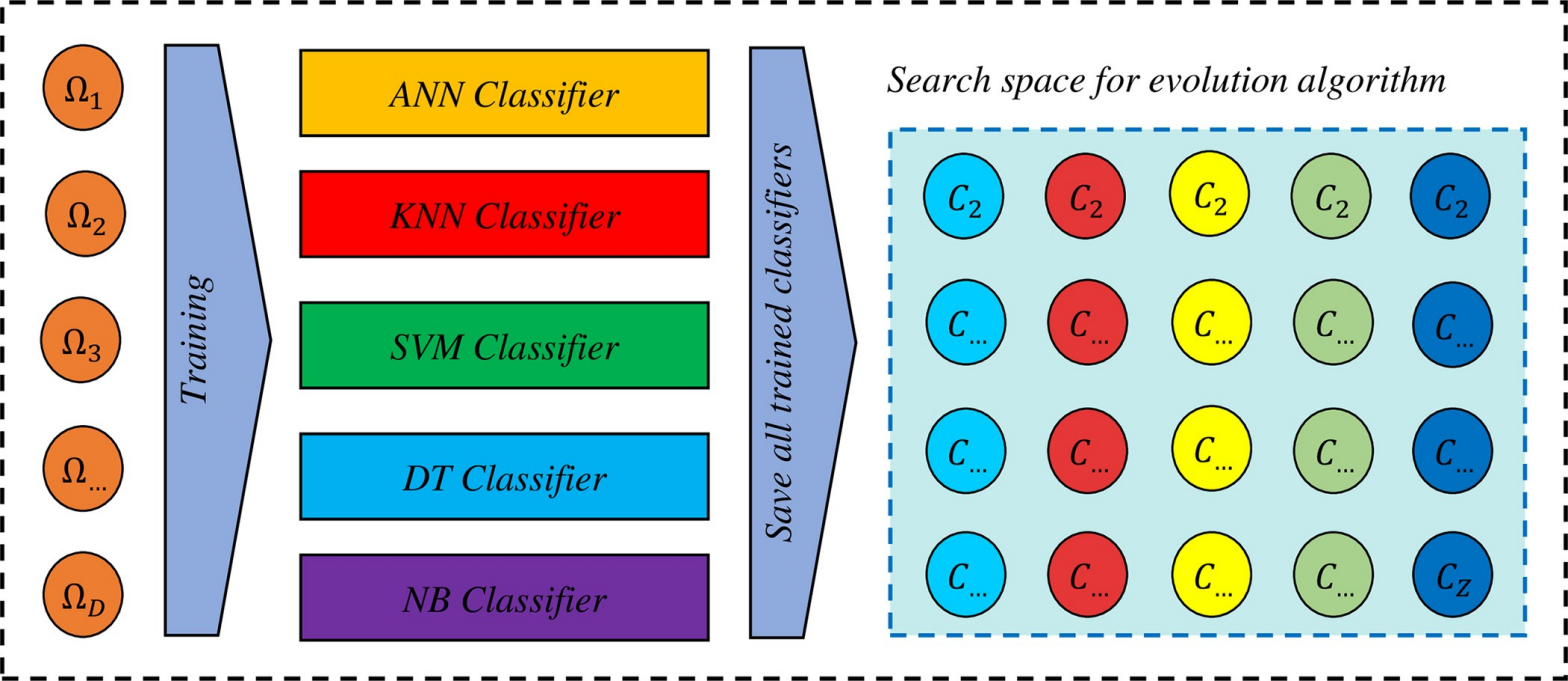
Creation of the evolutionary algorithm search space.

After removing the atomic and duplicate clusters, the number of D clusters remains, and the number of M classifiers are trained by each of the clusters. Accordingly, the total number of classifiers trained will be Z = D × M. Fig 4 illustrates the creation of the evolutionary algorithm search space.

#### The chromosome coding

**Fig 5:**
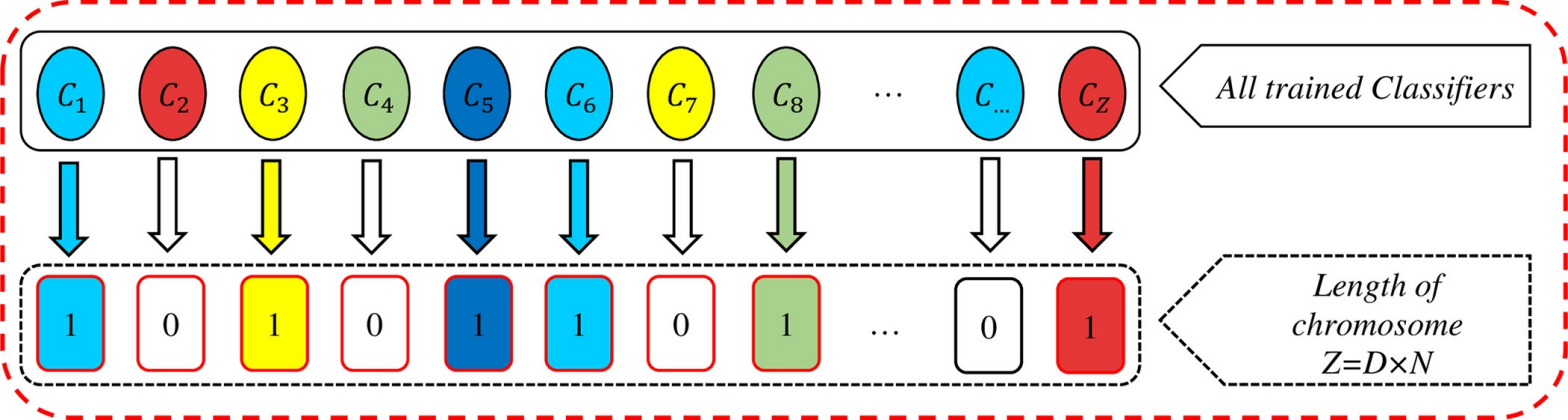
The chromosome representation in the proposed method.

In this study, the binary coding method is used. Each chromosome represents a possible ensemble model in which each gene is assigned to a unique base classifier. In order to maintain population diversity, chromosome initialization is performed randomly and is expressed as zero and one, in which zero indicates the absence of the corresponding classifier in the ensemble model and one indicates the presence of the corresponding classifier. Fig 5 shows an example of a chromosome (a feasible solution). It should be noted that the chromosome length in this case, is equal to the total number of trained classifiers in the search space (Z).

#### Formulation of the multi-objective problem

The problem of multi-objective optimization for the possible solution of E_i_ in the possible decision space (ε) can be defined as Eq (1):

$$\begin{array}{c} minimize(F(E_{i}))=minimize(f_{1}(E_{i}),f_{2}(E_{i}))\\ subject\;to\;E_{i}\in \varepsilon \end{array}$$ Eq (1)

Where f_j_ and E_i_ represent the j^th^ objective and the i^th^ set of classifiers, respectively. The ensemble classifier of solution i (E_i_) can be obtained from a combination of classifiers {C_1_, C_3_, C_5_, C_6_, C_8_, …, C_z_} by majority voting. Based on this, the output class of solution i is obtained through the following equation.


$$output\;class(E_{i})=mode(\{C_{1},C_{3},C_{5},C_{6},C_{8},\ldots ,C_{z}\})$$ Eq (2)

Where the *mode* function specifies the class that more classifiers have predicted it as the selected class. The objective functions are used to select the best ensemble classifier, which are different in each version of the proposed method. The two versions of the proposed method are called MOEEC-1 and MOEEC-2, respectively, which are described below.

*The first version of the proposed model (MOEEC_1)*. This version’s used objective functions include accuracy and the proposed diversity measure, which are described below.

#### *The first objective functi*o*n*

The objective *f*_*1*_ in this version which evaluates the classification performance of the ensemble model is Accuracy, calculated as Eq (3):

$$Accuracy=\frac{1}{n}\sum_{i=1}^{n}I_{i},I_{i}=\left\{ \begin{array}{c} 1\;\;\;\;if\;C_{i}=P_{i}\;\\ 0\;\;\;\;\;otherwise\; \end{array}\right.$$ Eq (3)

Where C_i_ is equal to the actual class of the i^th^ sample, P_i_ is the ensemble classifier prediction of the i^th^ sample, and n is the number of training samples. Since the goal in the proposed method is to minimize the values of the objective functions, 1-Accuracy is considered as the value of the objective function.

#### *The second object*i*ve function (the proposed diversity measure)*

The second objective function is the Diversity measure, which evaluates the diversity between the base classifiers of the ensemble classifiers. In [17], different metrics are introduced to assess diversity. These metrics calculate the diversity between two classifiers based on the difference in their classification error. This kind of calculation causes the difference between the two classifiers to be under-evaluated, and the ensemble model fails to select the classifiers that are quite different from each other. To resolve this problem, a novel diversity measure is proposed that evaluates the diversity of the two classifiers based on the differences between their predictions. Accordingly, if two classifiers have different predictions of a dataset, the proposed diversity measure will evaluate this difference. Consequently, if the X dataset contains N samples and the two classifiers C_i_ and C_j_ predict the samples of dataset X separately, then the difference between the predictions of these two classifiers can be obtained by Eq (4).

$${Div}_{C_{i},C_{j}}=\frac{1}{N}\sum_{k=1}^{N}f_{C_{i},C_{j}}(x_{k})$$ Eq (4)

Where the comparison function $f_{C_{i},C_{j}}(x_{k})$ is calculated as Eq (5):

$$f_{C_{i},C_{j}}(x_{k})=\left\{ \begin{array}{c} 1\;\;\;\;\;\;\;if\;\;\;\;c_{i}(x_{k})\neq c_{j}(x_{k})\\ 0\;\;\;\;\;\;\;if\;\;\;\;c_{i}(x_{k})=c_{j}(x_{k}) \end{array}\right.$$ Eq (5)

Where i and j are the classifiers’ indices, and k is the index of each data instance. The value 1 in this function indicates the difference between the two classifiers’ predictions of the corresponding sample, and the value 0 signifies their similarity in the predictions of that sample. Now, if we have an ensemble model containing L base classifiers, the diversity between its base classifiers is calculated by Eq (6).

$${Div}_{T}=\frac{2}{L(L-1)}\sum_{i=1}^{L-1}\sum_{j=i+1}^{L}{Div}_{C_{i},C_{j}}$$ Eq (6)

Since the goal in the proposed method is to minimize the values of the objective functions, ‘1−*Div*_*T*_’ is considered as the value of the objective function.

*The second version of the proposed model (MOEEC_2)*. The Performance evaluation index is a key factor in addressing the imbalance problem. Given the weakness of the Accuracy in evaluating classification performance in imbalanced data, in the proposed method as the second version, a new metric is used as the first objective function to evaluate the performance of the ensemble classifier, which is introduced below. Consider the database in Table 2, which includes the samples X = {x_1_, x_2_, …, x_10_}, and the three classes {a, b, c}.

**Table 2:** The dataset X including 10 samples and three classes a, b, and c.

*Samples*	*x*_1_	*x*_2_	*x*_3_	*x*_4_	*x*_5_	*x*_6_	*x*_7_	*x*_8_	*x*_9_	*x*_10_
*Classes*	*a*	*b*	*c*	*a*	*b*	*c*	*a*	*a*	*a*	*a*

Now, consider classifier C, which is predicted samples of dataset X, according to Table 3:

**Table 3:** The predictions of classifier C for samples of dataset X.

*Samples*	*x*_1_	*x*_2_	*x*_3_	*x*_4_	*x*_5_	*x*_6_	*x*_7_	*x*_8_	*x*_9_	*x*_10_
*Predictions*	*a*	*b*	*b*	*a*	*c*	*a*	*a*	*a*	*a*	*a*

The prediction results of classifier C can be rewritten, according to Table 4.

**Table 4:** The number of correct predictions in each class.

*Classes*	*Number of samples that* belong to each class (*R*_*i*_)	*Number of correct* Predictions in each class (*T*_*i*_)
*a*	6	6
*b*	2	1
*c*	2	0

Now, if we denote the number of classes in the dataset by *Num*_*c*_, the total number of samples related to the i^th^ class by *R*_*i*_, and the number of samples related to the *i*^th^ class that predicted correctly by classifier C indicated by T_i_, then the proposed metric can be calculated as Eq (7).

$$ImbalanceAccuracy=\frac{1}{{Num}_{C}}\sum_{i=1}^{{Num}_{C}}\left(\frac{T_{i}}{R_{i}}\right);i\in \{1,\ldots ,{Num}_{c}\}$$ Eq (7)

Which is actually the average of the classification accuracy in all classes. Accordingly, the value of this function for the above example is equal to $ImbalanceAccuracy=\frac{1}{3}\left(\frac{6}{6}+\frac{1}{2}+\frac{0}{2}\right)=0.5$.

This function causes the optimization algorithm to select a set of base classifiers from which the resulting ensemble model can classify samples of all classes. Since the goal is to minimize the value of the objective functions, *1-ImbalanceAccuracy* is considered as the value of the objective function. In this version of the proposed method, the second objective function is the proposed diversity measure.

## Section 4: Experimental framework

For evaluation of the churn prediction model in this study, a data set including contact information related to 3150 subscribers of an Iranian mobile service provider was used, which was collected over a period of 12 months. Table 5 briefly describes all 11 attributes of this dataset. Also, the ratio of churner customers in the data set is 15.7%, which indicates the unbalanced nature of this dataset [77].

**Table 5:** Features of dataset.

Row	Equivalent	Features of dataset	Main group
*1*	*Call failures*	Contacts that did not result in communication	Customer dissatisfaction
*2*	*Complains*	To complain	
*3*	*Subscription length*	The length of the customer relationship with the operator	
*4*	*Charge amount*	The amount of credit charged	Service usage level
*5*	*Seconds of use*	Call duration	
*6*	*Frequency of use*	Number of calls received	
*7*	*Frequency of SMS*	Number of text messages sent	
*8*	*Distinct called numbers*	Number of different phone numbers that the subscriber called with	
*9*	*Age group*	Age of subscriber	Customers’ features
*10*	*Tariff plan*	Type of service plan used by the subscriber	Variable costs
*11*	*Status*	Status of subscriber	Intermediate variable

During the performance evaluation process, the dataset is randomly divided into three parts, training dataset, evaluation dataset and test dataset, respectively, 70%, 10%, and 20%. The training dataset is used to train the base classifiers, then the evaluation dataset is used in the optimization algorithm to select the best classifiers combination. Finally, the test dataset is used to evaluate the performance of the selected ensemble. This process is performed five times, and the numbers reported in this study are the average of five iterations.

### Parameters tuning

The first parameter to be specified is the number of clustering known as the ‘upper bound of clustering’ represented by *n*. The relationship between *n* and the total number of produced clusters after clustering is p = n (n + 1)/2, so if the value of *n* is considered a large value, the number of clusters produced will be very large, which causes computational complexity. Hence the value of this parameter is determined through trial-and-error. The value of this parameter in this study is 10, in which case, the total number of clusters produced will be 55.

The proposed method has been implemented in the MATLAB programming language version 2019. For training on each cluster, a set of five different classifiers are used, including artificial neural networks, support vector machines, K nearest neighbors, decision trees, and Naive Bayes. The parameters used in the multi-objective evolutionary algorithm are determined by trial-and-error. The selection of individuals done by the Tournament selection method; for recombination operator in evolutionary algorithm three functions of single-point, double-point, and uniform crossover with equal probability were used. The mutation function randomly mutates 3% of the genes. Table 6 shows the parameters used in clustering algorithms, base classifiers and evolutionary algorithms in the proposed method.

**Table 6:** Tuned parameters of algorithms.

Algorithm	Parameter
ANN	Default
KNN	Distance metric = Euclidean; Number of nearest neighbors = 10; Distance weighting function = squared inverse; Standardization = true
SVM	Kernel function = Polynomial; Polynomial kernel function order = 3; Standardization = true
DT	Split criterion = Gini’s diversity index; Maximal number of decision splits = 100; Surrogate = off
NB	Data distributions = Multinomial distribution.
K-means	Max iterations = 2400; Distance measurement = Squared Euclidean
NCA	Solver = stochastic gradient descent; Fit method = exact; Standardization = true
NSGA-II	Length of chromosome = all trained classifiers; population size = 100; generation = 130; crossover probability = 0.7; mutation probability = 0.4

### The performance evaluation indicators

To evaluate the performance of the proposed model and other ensemble learning methods, the following seven performance evaluation criteria are used, including Accuracy, AUC, Recall, Specificity, Precision, F-score, and G-means. Consider a two-class problem in which the minority class to be a positive class and the majority class to be a negative class. In that case, the confusion matrix for it can be represented as in Table 7. *P* is the number of positive class samples (minority class), and *N* is the number of negative class samples (majority class).

**Table 7:** The confusion matrix.

		Predicted classes		Total
		*Positive class (minority)*	*Negative class (majority)*	
Real Classes	*Positive class (minority)*	True positive (TP)	False negative (FN)	*P*
	*Negative class (majority)*	False positive (FP)	True negative (TN)	*N*
Total		*P*′	*N*′	*P*+*N*

In Table 7, TP, TN, FP, and FN represent the positive class samples that are correctly classified, the negative class samples that are correctly classified, the negative class samples that are incorrectly classified, and the positive class samples that are classified as negative. According to the confusion matrix in Table 7, the values of each of the seven indicators are calculated by Eqs (9) to (15).


$${FP}_{rate}=\frac{FP}{N}\}$$ Eq (8)

$$Accuracy=\frac{TP+TN}{P+N}$$ Eq (9)

$$AUC=\frac{1+{TP}_{rate}-{FP}_{rate}}{2}$$ Eq (10)

$${TP}_{rate\;}or\;recall=\frac{TP}{P}$$ Eq (11)

$${TN}_{rate}\;or\;Specificity=\frac{TN}{N}$$ Eq (12)

$$Precision=\frac{TP}{TP+FP}$$ Eq (13)

$$G-mean=\sqrt{\frac{TP}{P}\times \frac{TN}{N}}$$ Eq (14)

$$F-score=\frac{Precision\times Recall\times 2}{Precision+Recall}$$ Eq (15)

### The experimental results

In this section, the results of the proposed method on the churn dataset, along with a comparison of its performance against some of the most recent classification algorithms in the literature, are presented. The following is a description of the optimization steps on the churn dataset. After that, a comparison is made between the proposed algorithm and other ensemble models, classical classifiers, and the results related to previous research in terms of Accuracy, AUC, Recall, Precision, Sensitivity, F-score and G-means.

#### Illustration of MOEEC

**Fig 6:**
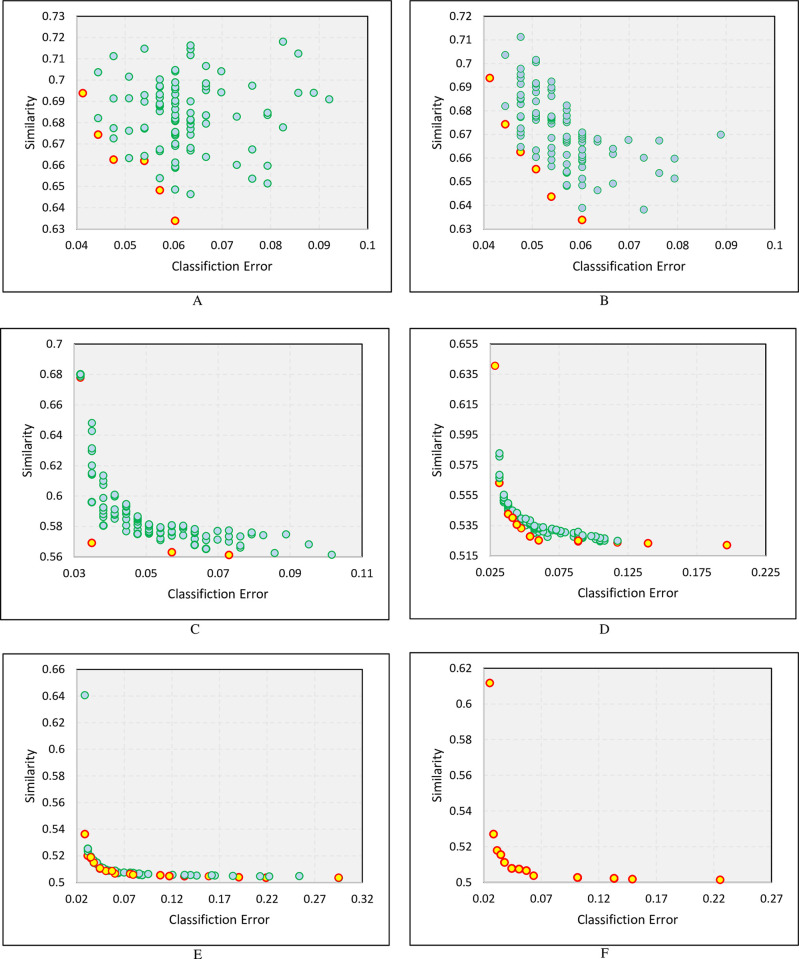
Illustrating the set of non-dominated solutions in different generations with respect to the two objectives, ‘accuracy’, and ‘diversity’.

Fig 6 shows the evolution of population into six different generations in the MOEEC-1 algorithm. Each individual is an ensemble model consisting of several base classifiers that have been experimentally selected from the search space’s classifiers. The combination of these classifiers by majority voting; Obtains an ensemble classifier for which the values of the objective functions are represented in two dimensions. The X-axis in the diagrams in Fig 6 presents the value of the first objective function (1−accuracy) or the classification error, and the Y-axis represents the values of the second objective function (1−diversity) or the similarity between the base classifiers. Accordingly, with decreasing the value of objective functions, the value of Accuracy and Diversity will increase. Different combinations of base classifiers will create different ensemble models in which the values of accuracy and diversity are different for each one. The ensemble models located on the Pareto-front are the most desirable ones.

Yellow circles indicate non-dominated ensemble models in Fig 6 at each stage. These non-dominated solutions have performed better than others in terms of accuracy and diversity. Fig 6 illustrates how these non-dominated solutions get better with each successive generation. The first generation of the population (diagram A in Fig 6) consists of chromosomes with random combinations of genes, in which the best value of accuracy is about 96% and the best values of diversity is about 47%. However, in subsequent generations, the algorithm will move towards finding the best combination of genes (base classifiers). Finally, in diagram F in Fig 6, the Pareto front is shown with its best possible combination regarding the two objectives of Diversity and Accuracy.

**Fig 7:**
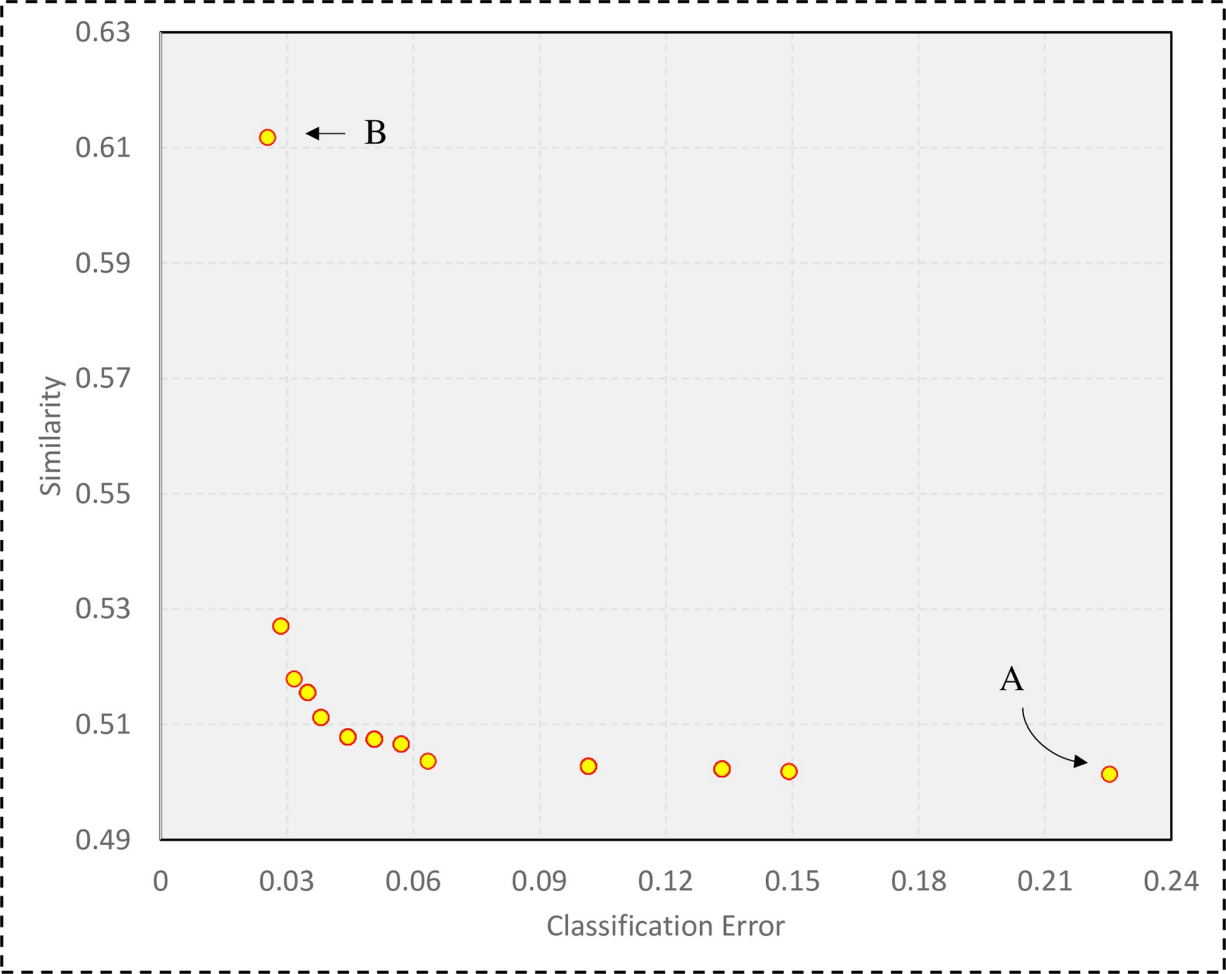
The final population of the optimization algorithm based on the two goals of accuracy and diversity.

Fig 7 illustrates the final population of the optimization algorithm. According to Fig 7, the created ensemble models are in the best possible range based on the two functions of classification error and similarity. Although the ensemble model A does not perform well in terms of classification accuracy, the value of diversity between its base classifiers is close to 50%. While the ensemble model B has a good performance in terms of classification accuracy and close to 97%, its base classifiers have a diversity of nearly 39%. In this study, in order to more accurately identify the customers, the selected ensemble model with better classification accuracy has been selected. Hence, the MOEEC_1 model is actually the ensemble model B.

**Fig 8:**
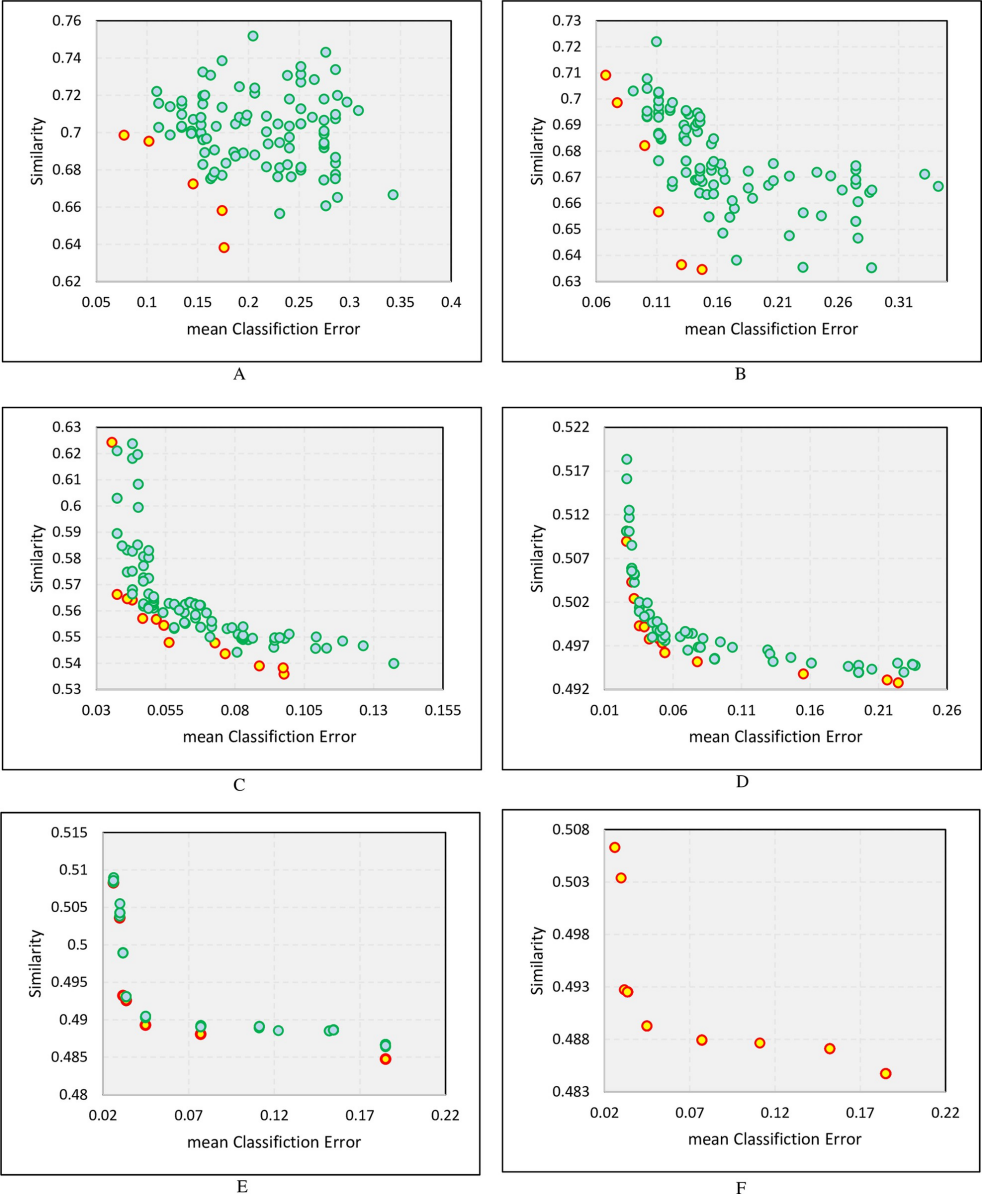
The set of non-dominated solutions in different generations with respect to two objectives diversity and imbalance accuracy.

In this study, in order to overcome the imbalance problem, as the second version of the proposed model, instead of Accuracy, a new objective function has been used to select a set of base classifiers, which the combination of them by majority voting results in an ensemble model, whose classification performance is optimal in both churn and non-churn classes. Fig 8 shows the results of using this function in the proposed model. The X-axis in Fig 8 represents the values of the first objective function (1-ImbalanceAccuracy) or the mean of the classification error, and the Y-axis represents the values of the second objective function (1-diversity) or the similarity amount between the base classifiers. Accordingly, as the values of the objective functions are minimized, the values of Imbalance Accuracy and Diversity increase.

The non-dominated ensemble models in the diagrams of Fig 8 at each stage are indicated by yellow circles. The first generation of the population (diagram A in Fig 8) consists of chromosomes with a combination of random genes, in which the best value of Imbalance Accuracy is about 93%, and the best value of Diversity is about 56%. However, in subsequent generations, the algorithm will move towards finding the best combination of genes (base classifiers). The Pareto front of the last figure (diagram F in Fig 8) illustrates the best possible combination of base classifiers with respect to the two goals of Diversity and Imbalance Accuracy.

**Fig 9:**
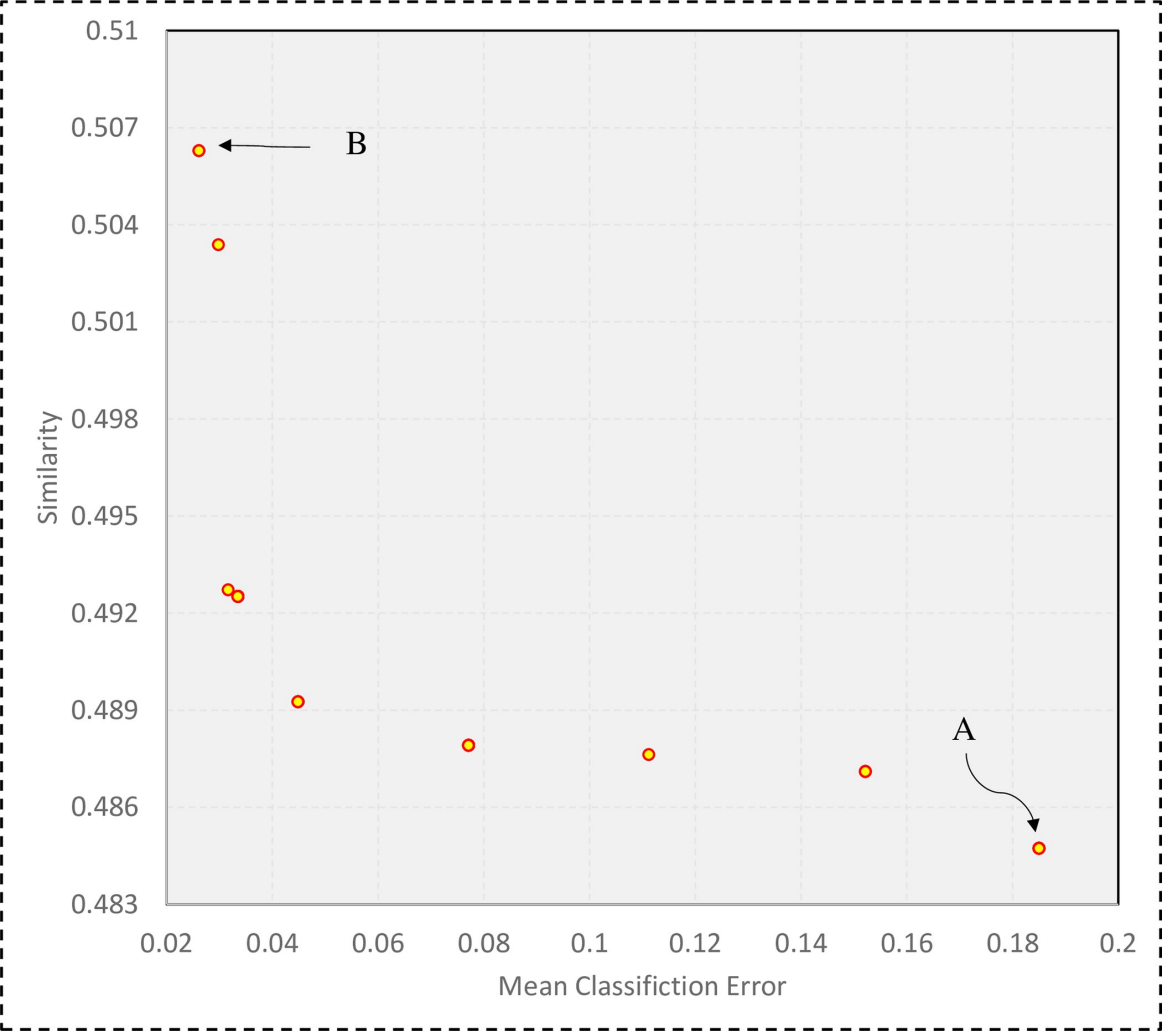
The final population of the optimization algorithm based on the two goals of imbalance accuracy and diversity.

Fig 9 shows the ensemble models created in the last generation of the algorithm. Based on two objective functions, mean of classification error and similarity, the created ensemble models are in the best possible range. Based on this information, although ensemble model A, is not a good performer in terms of Imbalance Accuracy, the diversity of its base classifiers is close to 52%. While the ensemble model B has a desirable performance in terms of imbalance accuracy (close to 98%), and its base classifiers have a diversity of nearly 50%. Since this study aims to identify customer churn more accurately, we chose an ensemble model with higher classification accuracy. Hence the MOEEC_2 model is the ensemble model B.

#### Comparison

In this section, the results of the two proposed models in this paper have been first compared with the five classical classifiers, ANN, KNN, DT, SVM, NB, LR, and then with some ensemble models in the literature. Table 8 shows values of the seven performance evaluation metrics, Accuracy, AUC, Recall, Specificity, Precision, F-score, and G-means for classical algorithms. Each row represents the values of the indicators related to each model. In Table 8, in each index column, the values related to the three best models are highlighted.

**Table 8:** Comparison of the proposed algorithms with classical classifiers.

Num	Classifier	Accuracy	AUC	Recall	Specificity	Precision	F-score	G-means
1	*MOEEC-1*	**97.30**	**93.77**	**88.66**	**98.87**	**93.48**	**91.00**	**93.63**
2	*MOEEC-2*	**96.35**	**94.89**	**92.78**	96.99	84.91	**88.67**	**94.86**
3	*ANN*	94.38	86.89	75.96	97.82	**86.64**	80.95	86.19
4	*KNN*	94.98	89.29	81.01	97.59	**86.24**	83.54	88.91
5	*DT*	**95.14**	**90.96**	84.85	97.06	84.34	**84.59**	**90.75**
6	*SVM*	89.56	69.07	39.19	**98.96**	87.39	54.11	62.27
7	*Naïve Bayes*	74.57	81.29	**91.11**	71.49	37.33	52.97	80.71
8	*Logistic regression*	89.17	70.89	44.24	**97.55**	77.11	56.23	65.69

**Fig 10:**
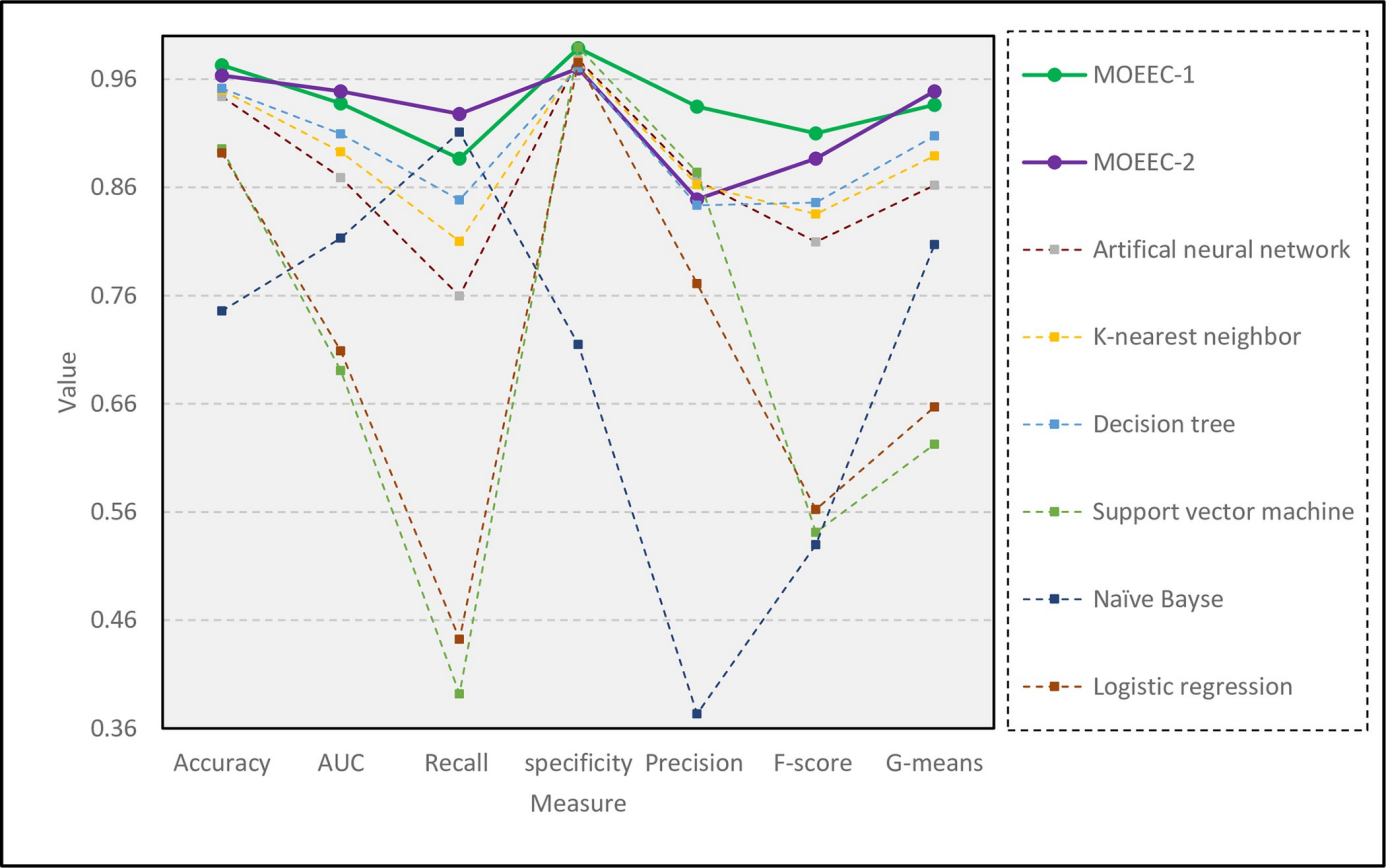
Comparison of the proposed models with classical classifiers.

Table 8 shows that the proposed models outperform all classical algorithms in terms of five measures Accuracy, AUC, Recall, F-score, G-means. Also, the MOEEC-1 model is one of the top three models in two indicators ‘Specificity’ and ‘Precision’. Besides, Fig 10 shows the performance of the compared algorithms. Therefore, in each index, the algorithm that has the best and weakest performance can be identified separately.

Table 9 also presents the values of seven performance evaluation metrics to compare the proposed models with some existing ensemble models in the ensemble learning literature. In Table 9, the values related to the three best models are highlighted in each index column.

**Table 9:** Comparison of the proposed algorithms with other ensemble models.

Num	Classifiers	Accuracy	AUC	Recall	Specificity	Precision	F-score	G-means
1	*MOEEC-1*	**97.30**	**93.76**	**88.66**	**98.87**	**93.48**	**91.01**	**93.63**
2	*MOEEC-2*	**96.35**	**94.89**	**92.78**	96.99	84.90	**88.67**	**94.87**
3	*Subspace Discriminate*	89.90	72.32	40.40	**99.13**	**89.68**	55.71	63.29
4	*Bagging*	94.95	89.28	81.01	97.55	86.05	83.45	88.89
5	*AdaBoost_j48*	95.62	90.82	83.84	97.81	87.74	85.74	90.56
6	*Stacking*	95.14	90.95	84.85	97.06	84.34	84.59	90.75
7	*Random Forest*	**96.13**	91.21	84.04	**98.38**	**90.63**	87.21	90.92
8	*Ensemble Selection*	94.67	89.85	82.82	96.87	83.16	82.99	89.57
9	*Random Committee*	95.49	89.52	80.81	98.23	89.48	84.92	89.09
10	*Random Subspace*	94.41	86.82	75.76	97.89	87.00	80.99	86.11
11	*Rotation Forest*	96.09	**92.01**	**86.06**	97.96	88.75	**87.38**	**91.82**

**Fig 11:**
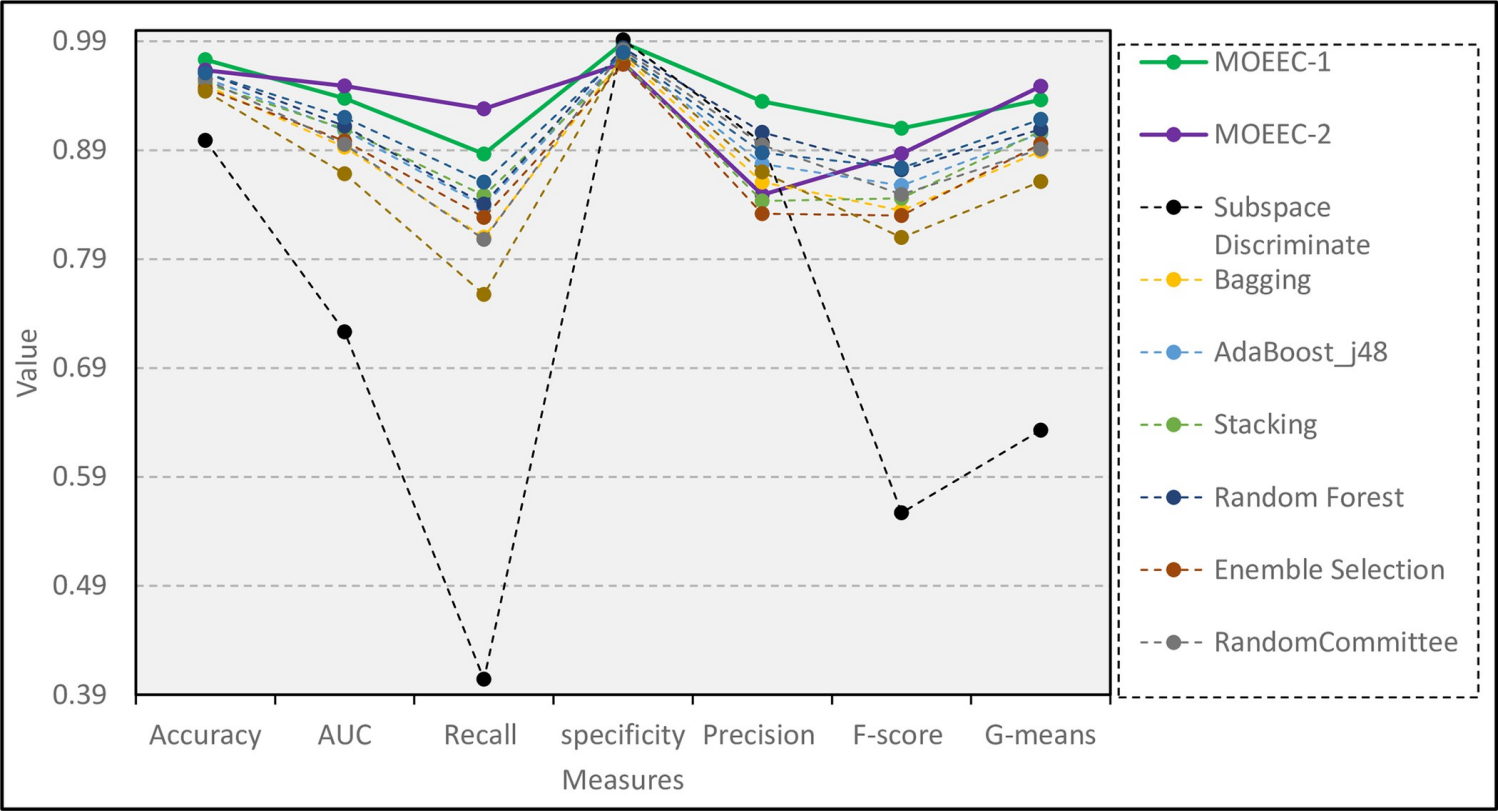
Comparison of the proposed models with other ensemble models.

As shown in Table 9, the two presented models in this paper outperformed other ensemble models in the five metrics of Accuracy, AUC, Recall, F-score, and G-means. According to Tables 8 and 9, it can be concluded that in Accuracy, MOEEC-1, MOEEC-2 and Random Forest models had better performance than other models, with values of 97.30, 96.35 and 96.13, respectively. Also, in AUC, the three models MOEEC-2, MOEEC-1 and Rotation Forest performed better than the other models, with values of 94.89, 93.76 and 92.01, respectively. Fig 11 compares the proposed models with other ensemble models in terms of performance evaluation metrics.

#### Comparison with the existing churn models

In this section, the proposed models are compared with the churn models presented in previous studies that have used the dataset of this research. In [3], the researchers presented a hybrid model consisting of four classifiers DT, ANN, KNN, and SVM, combined by a ranking method. In this method, the researchers use a control variable with values between 0.5 and 2.2 to tune the model, which yields different Recall, Precision, and F-score values. Therefore, to compare the values of these metrics reported by the researchers, the averages are considered and then compared with the values obtained in this research. In [77], researchers presented a model developed by neural networks (ChP-SOEDNN) that uses two self-organizing and error-driven learning approaches. Table 10 compares the values reported in the previous two studies with the values obtained from the experiment in this study.

**Table 10:** Comparison of the proposed two models with the presented models in the literature.

Model	Accuracy	Recall	Precision	F-score
*MOEEC-1*	**97.30**	88.66	**93.48**	**91.005**
*MOEEC-2*	**96.35**	**92.78**	**84.90**	**88.67**
*Hybrid method [3]*	Not reported	65.865	84.8875	67.8025
*ChP-SOEDNN [77]*	95.13	**95.71**	77.01	85.71

According to Table 10, it can be seen that MOEEC-1 and MOEEC-2 performed better than the models presented in the literature in three measures of Accuracy, Precision, and F-score.

## Section 5: Conclusion

In today’s competitive market, customer churn presents a formidable challenge for businesses, particularly in the telecommunications sector. Our study has focused on developing advanced churn prediction models leveraging ensemble learning, layered clustering, and multi-objective optimization techniques. The results demonstrate the efficacy of our proposed models, with superior performance across key metrics such as Accuracy, AUC, F-score, and G-means. Specifically, the MOEEC-1 model exhibits dominance in Accuracy, Precision, and F-score, while the MOEEC-2 model excels in AUC, Recall, and G-means metrics. Moreover, our study introduces novel evaluation metrics for measuring diversity and classification performance, contributing to the refinement of churn prediction methodologies.

Looking ahead, it is essential to acknowledge the limitations of our approach and identify avenues for future research. Future studies could explore the integration of additional data sources and the refinement of feature engineering techniques to enhance model performance. Additionally, as the telecommunications landscape continues to evolve, there is a need for adaptive churn prediction methodologies that can accommodate emerging trends and technologies. By embracing interdisciplinary approaches and continually refining predictive models, researchers can empower businesses with actionable insights to mitigate customer churn and foster long-term relationships in an ever-changing market environment.

In conclusion, while our study represents a significant step forward in the domain of churn prediction, there remains ample room for further exploration and refinement. While our findings showcase promising results across various performance metrics, it is essential to recognize the inherent limitations of our approach. One notable limitation is the reliance on historical data, which may not fully capture evolving customer behaviors and market dynamics. Furthermore, the interpretability of complex ensemble models remains a challenge, hindering actionable insights for businesses. By embracing interdisciplinary approaches and staying attuned to industry dynamics, researchers can continue to innovate and develop predictive models that empower organizations to thrive in an ever-evolving marketplace. Also, future research endeavors could explore the integration of real-time data streams and dynamic modeling techniques to enhance the adaptability and robustness of churn prediction models.

## Supporting information

S1 Link Source code repository. In this link, you can find the source code for all formulas and Algorithms.
(DOCX)

S1 File (DOCX)
