# Supplementary material for: Customer churn modeling in telecommunication using a novel multi-objective evolutionary clustering-based ensemble learning
Source: PLoS One. 2024 Jun 6;19(6):e0303881. doi: 10.1371/journal.pone.0303881 (PMC11156398; doi:10.1371/journal.pone.0303881)
Supplement: S1 Link — In this link, you can find the source code for all formulas and Algorithms. (DOCX) [file pone.0303881.s001.docx]

S1 Link. Source code Repository:

<https://github.com/kavehfaraji/thesis-Source-code>.
