# Supplementary material for: Customer churn modeling in telecommunication using a novel multi-objective evolutionary clustering-based ensemble learning
Source: PLoS One. 2024 Jun 6;19(6):e0303881. doi: 10.1371/journal.pone.0303881 (PMC11156398; doi:10.1371/journal.pone.0303881)
Supplement: S1 File — (DOCX) [file pone.0303881.s002.docx]

Dear Dr. Anwar,

Thank you for bringing PLOS ONE's guidelines on code sharing to our attention. We appreciate the emphasis on transparency and reproducibility in research.

We have ensured that all author-generated code underpinning the findings in our manuscript is available without restrictions. Our code repository can be accessed via the following link: <https://github.com/kavehfaraji/thesis-Source-code>.

We believe that sharing our code in this manner aligns with best practices and facilitates reproducibility and reuse. Should you require any further information or assistance, please do not hesitate to contact us.

Regarding the information about our colleague who edited our manuscript, I must inform you that Mr. Kaveh Faraji Googerdchi edited our manuscript.

Thank you for your attention to this matter.

Sincerely,
